# Reply to Ngoh et al. Comment on “Surendran et al. The Missed Opportunity of Patient-Centered Medical Homes to Thrive in an Asian Context. *Int. J. Environ. Res. Public Health* 2021, *18*, 1817”

**DOI:** 10.3390/ijerph19084686

**Published:** 2022-04-13

**Authors:** Shilpa Surendran, Chuan De Foo, Chen Hee Tam, Elaine Qiao Ying Ho, David Bruce Matchar, Josip Car, Gerald Choon Huat Koh

**Affiliations:** 1 Health Systems and Behavioral Sciences Domain, Saw Swee Hock School of Public Health, National University Singapore, 12 Science Drive 2, Singapore 117549, Singapore; ephfchu@nus.edu.sg (C.D.F.); ephtamc@nus.edu.sg (C.H.T.); hoqiaoying@gmail.com (E.Q.Y.H.); ephkohch@nus.edu.sg (G.C.H.K.); 2 Health Services and Systems Research, Duke—NUS Medical School, 8 College Road, Singapore 169857, Singapore; david.matchar@duke-nus.edu.sg; 3 Department of Medicine (General Internal Medicine), Duke University School of Medicine, 400 Morris Street 3rd Floor, Durham, NC 27701, USA; 4 Centre for Population Health Sciences, Lee Kong Chian School of Medicine, Nanyang Technological University, 11 Mandalay Road, Singapore 308232, Singapore; josip.car@ntu.edu.sg

We carefully read the comment [1] written to the editor referring to our original article titled “The Missed Opportunity of Patient-Centered Medical Homes to Thrive in an Asian Context” [2], and we are thankful for the opportunity to reply to the comment. 

It was stated in the comment that the Ang Mo Kio Family Medicine Clinic (AMK FMC), the local version of Patient-Centered Medical Home (PCMH), was not represented in the study. However, we had approached all the PCMHs in Singapore including the AMK FMC, but we were unsuccessful in recruiting potential participants from the AMK FMC, which is mentioned in our article. Nevertheless, we are glad to have information about AMK FMC through the comment.

The objective of our study was to identify the contextual healthcare policy factors that influenced the implementation of PCMHs in Singapore. Hence, we can only comment on the aspects related to this. Ngoh et al. [1] stated that the health outcomes of the patients transferred to AMK FMC from National Healthcare Group polyclinics have remained satisfactory. However, this is beyond our study’s scope, and we do not have this data from other PCMHs to comment upon.

The challenges experienced by the AMK FMC, i.e., disruption in care continuity when patients exhausted their quantum of yearly subsidies, is interesting and supports the findings from our study. 

We are heartened to read that AMK FMC has been a financially viable PCMH. It is an exception and it required huge commitment from their partners with other unique features. Variations in terms of leadership composition and hence the willingness to share decision-making, level of financial backing from privately partnered organizations post-privatization and extent of horizontal and vertical integration agreements that permit the transfer of patients from polyclinics and tertiary hospitals respectively to PCMHs differ across PCMHs and also change over time to list a few. Hence, within the remit of our article, we can only comment on the data that we obtained from seven out of the nine PCMHs in Singapore and within the timeframe of data consolidation. The issues raised in our article remain broadly relevant, regardless of the PCMH in question, and should be promoted when exploring similar facilities going forward.
